# Anesthetic Management Recommendations Using a Machine Learning Algorithm to Reduce the Risk of Acute Kidney Injury After Cardiac Surgeries

**DOI:** 10.5812/aapm-143853

**Published:** 2024-06-05

**Authors:** Ahmad Ali Abin, Ahmad Molla, Azar Ejmalian, Shahabedin Nabavi, Behnaz Memari, Kamal Fani, Ali Dabbagh

**Affiliations:** 1 Faculty of Computer Science and Engineering, Shahid Beheshti University, Tehran, Iran; 2 LIFAT, Universite de Tours, Tours, France; 3 Department of Anesthesiology, School of Medicine, Iran University of Medical Sciences, Tehran, Iran; 4 Anesthesiology Research Center, Shahid Beheshti University of Medical Sciences, Tehran, Iran; 5 Department of Anesthesiology, School of Medicine, Shahid Beheshti University of Medical Sciences, Tehran, Iran

**Keywords:** Acute Kidney Injury, Cardiac Anesthesia, Machine Learning

## Abstract

**Background:**

Open heart surgeries are a common surgical approach among patients with heart disease. Acute kidney injury (AKI) is one of the most common postoperative complications following cardiac surgeries, with an average incidence of 6 - 10%. Additionally, AKI has a mortality rate of 5 - 10%. One of the challenges of cardiac surgeries is selecting the appropriate anesthetic approaches to reduce the risk of AKI.

**Objectives:**

This study presents a machine learning-based method that consists of two regression models. These models can inform the anesthesiologist about the risk of AKI resulting from the improper selection of anesthetic parameters.

**Methods:**

In this cohort study, the medical records of 998 patients who underwent cardiac surgery were collected. The proposed method includes two regression models. The first regression model recommends optimal anesthesia parameters to minimize the risk of AKI. The second model provides the anesthesiologist with the safest margin for deciding on anesthetic parameters during surgery, including cardiopulmonary bypass (CPB) time, anesthesia time, crystalloid dose, diuretic dose, and transfusion of packed red cells (PC) and fresh frozen plasma (FFP). Using this method, the specialist can evaluate the anesthetic parameters and assess the potential AKI risk. Additionally, the proposed method can also provide the treatment team with anesthetic parameters that carry the lowest risk of AKI.

**Results:**

This method was evaluated using data from 526 patients who suffered from postoperative AKI (AKI+) and 472 who did not suffer any injury (AKI-). The accuracy of the proposed method is 80.6%. Additionally, the evaluation of the proposed method by three experienced cardiac anesthesiologists shows a high correlation between the results of the proposed method and the opinions of the anesthesiologists.

**Conclusions:**

The results indicated that the outputs of the proposed models and the designed software could help reduce the risk of postoperative AKI.

## 1. Background

The rate of cardiac surgeries is increasing due to a rise in the average age of populations in communities and the need to address complications of aging, including cardiovascular disease (1). Open heart surgeries are complex and time-consuming procedures (2). Additionally, acute kidney injury (AKI) is one of the most critical postoperative complications that can occur after heart surgery for various reasons, including a higher possibility of AKI risk factors in patients undergoing cardiac surgeries (3).

Risk factors for AKI include the coexistence of conditions such as diabetes, hypertension, and advanced age (4). Furthermore, other factors leading to AKI are exclusively related to anesthesia, surgical procedures, and patient management in the intensive care unit. The unique aspects of heart surgeries closely related to this complication include the use of a cardiopulmonary pump, aortic cross-clamp, and large-volume blood transfusions (5). Acute kidney injury is characterized by an increase in serum creatinine, a decrease in absolute urine output, or both, based on which three different definitions of AKI have been developed (Table 1) (5).

**Table 1.** Patients Characteristics Before Cardiac Surgery ^a^

Variables	Values
**Age ** ^**b**^	57.44 ± 11.40 (18 - 85)
**Patient specific features**	
**Gender**	598 (59.92)
Male	
Female	400 (40.08)
**BMI ** ^**b**^ ** (kg/m** ^**2**^ **)**	26.76 ± 3.04 (17 - 41)
**Surgery**	
CABG	653 (65.43)
VALVULAR	177 (17.73)
TRANSPLANT	16 (1.60)
AORTIC surgeries	55 (5.51)
REDO	1 (0.10)
Mixed types	90 (9.01)
Missing values	6 (0.62)
**Type**	
Elective	825 (82.66)
Emergency	171 (17.13)
Missing values	2 (0.21)
**Past Medical History (PMH)**	
**HTN**	
Positive	715 (71.64)
Negative	283 (28.36)
**DM**	
Positive	509 (51)
Negative	488 (48.89)
Missing values	1 (0.11)
**CKD**	
Positive	154 (15.43)
Negative	843 (84.46)
Missing values	1 (0.11)
**PHTN**	
Positive	425 (42.58)
Negative	570 (57.11)
Missing values	3 (0.31)
**COPD**	
Positive	171 (17.13)
Negative	827 (82.87)
**Stenting**	
Positive	323 (32.36)
Negative	675 (67.64)
**CVA**	
Positive	122 (12.03)
Negative	878 (87.97)
**3VD**	
Positive	650 (65.13)
Negative	348 (34.87)
**Drug History (DH)**	
**ACEI**	
Positive	439 (43.98)
Negative	559 (56.02)
**ARB**	
Positive	393 (39.37)
Negative	600 (60.12)
Missing values	5 (0.51)
**BB**	
Positive	816 (81.76)
Negative	182 (18.24)
**Diuretics**	
Positive	608 (60.92)
Negative	390 (39.08)
**CCB ** ^**b**^	
Positive	291 (29.15)
Negative	707 (70.85)
**Statin**	
Positive	880 (88.17)
Negative	112 (11.22)
Missing values	6 (0.61)
**ASA**	
Positive	894 (89.57)
Negative	105 (10.43)
**NSAIDs**	
Positive	96 (9.61)
Negative	902 (90.39)
**Laboratory Parameters (LAB)**	
**EF**	48.05 ± 9.54 (10 - 66)
**Cr ** ^**b**^ ** (mg/dL)**	1.24 ± 0.80 (0.5 - 12)
**Alb ** ^**b**^ ** (g/dL)**	3.85 ± 0.66 (1.8 - 6.8)
**BS ** ^**b**^ ** (mg/dL)**	162.03 ± 53.4 (77 - 425)
**HbA1C ** ^**b**^ ** (mmom/mol)**	6.16 ± 1.19 (3.7 - 12)
**Hct ** ^**b**^	39.34 ± 4.10 (23 - 56)

Abbreviations; BMI, Body Mass Index; CABG, coronary artery bypass grafting surgery; PMH, past medical history; HTN, hypertension; DM, diabetes mellitus; CKD, chronic kidney disease; PHTN, pulmonary hypertension; COPD, chronic obstructive pulmonary disease; CVA , cerebrovascular accident; 3VD, three vessels disease; DH, drug history; ACEI, angiotensin convertase enzyme inhibitor; ARB, angiotensin receptor blocker; BB, beta blocker; CCB, calcium channel blocker; ASA, acetylsalicylic acid; NSAIDs, nonsteroidal anti-inflammatory drugs; EF, ejection fraction; Cr, creatinine; Alb, albumin; BS, blood sugar; Hb A_1_C, glycated hemoglobin; Hct, hematocrit.

^a^ Values are expressed as No. (%) or mean ± SD (range).

^b^ Selected features.

In coronary artery bypass grafting (CABG) surgery, a cardiopulmonary bypass pump is attached to the patient's main arteries and veins to act as the heart and lungs, delivering blood to different body parts and removing carbon dioxide from the blood. However, when the machine connects to the heart's arteries, blood flow to other parts of the body briefly stops, potentially reducing the glomerular filtration rate (GFR) of the blood and leading to postoperative AKI (6). Other factors contributing to AKI during cardiopulmonary bypass include changes in kidney vascular tone, blood interaction with artificial surfaces, and activation of the systemic inflammation cascade (5).

The average incidence of AKI after heart surgery is between 6 - 10%, with a corresponding mortality rate of 5 - 10% (6). However, a 2019 study reported this rate as high as 30% (5). About 3.1% of patients with postoperative AKI in recent years have required dialysis (7). The need for dialysis and renal replacement therapy (RRT), long-term hospitalization, and increased mortality rates are significant long-term consequences of AKI (5). The highest mortality rate due to kidney injury occurs in those over the age of 66, with an average rate globally in 2019 of about 6% (7).

Machine learning has been increasingly utilized in medical procedures across various fields (8, 9), and its applications in anesthesiology are expanding (10). Several studies have explored the use of artificial intelligence and machine learning methods to predict postoperative kidney injury. Rank et al. (11) introduced a deep learning method for predicting kidney injury after heart surgery. In this method, 96 important medical data points were collected from 15,564 patients, and the recurrent neural network (RNN)-based model achieved an area under the curve (AUC) of 0.893, significantly higher than the clinical accuracy of 74.5%. Additionally, the preoperative data were sequenced, and the time series method along with RNN was used to predict postoperative kidney injury. Furthermore, Thottakkara et al. (12) achieved an AUC of about 0.858 - 0.797 using logistic regression and support vector machine (SVM) methods on over 50,000 samples, demonstrating that the model performs well in predicting the occurrence of postoperative kidney injury. Moreover, Wei et al. (13) assessed the increasing severity of kidney injury based on logistic regression models and extreme gradient boosting (XGBoost) using data from 25,711 patients. Kidney injury severity is expressed as an integer from 0 - 3, where zero indicates no injury and three indicates the most severe injury. The following sections will detail how the severity of kidney injury is determined. Wei et al. identified patients whose kidney injury severity increased from stage 1 and 2 to stage 3 over time. Using the XGBoost model on the problem data (13), achieved higher accuracy than the logistic regression model (AUC = 0.926).

Research in this field is divided into predicting the occurrence or non-occurrence of kidney injury after cardiac surgery and the severity of kidney injury among patients over time. In the first category, the primary purpose of the studies is to predict whether kidney injury occurs after heart surgery. Koyner et al. achieved an AUC of 0.90 in predicting level 2 kidney injury (14). In another study, Tseng et al. designed various models such as logistic regression, random forest, SVM, XGboost, and ensemble learning on data from 671 patients. Ultimately, the Ensemble model (RF + XGboost) was selected as the best method for predicting postoperative AKI, achieving an AUC of 0.843 (15). Mohamadlou et al. used ensemble boosted decision trees to design a model based on data from over 300,000 samples, which achieved an AUC of 0.8 (0.809-0.792) (16). In another study, Lee et al. used feature selection methods and machine learning algorithms to obtain different accuracy values for the postoperative AKI prediction problem, among which the logistic regression method, based on data from 889 patients, achieved an AUC of 0.93 using the Monte Carlo cross-validation method (17, 18). Furthermore, Shawwa et al. applied a Gradient Boost method on data from 98,472 patients who underwent heart surgery from 2005 to 2017, achieving an AUC of 0.690 (0.697 - 0.682). The constructed model included 30 patients' preoperative and intraoperative features (19).

The second category of studies examines the increasing severity of kidney injury in patients over time. 

## 2. Objectives

In the current study, the severity of the patient's kidney injury on the first and seventh days after heart surgery was determined by measuring the patient's blood creatinine (Cr) levels and comparing them with preoperative levels. Considering the registered preoperative features of the patient, intraoperative anesthesia parameters, and the severity of the patient's kidney injury on the first and seventh days post-surgery, this study aims to suggest intraoperative anesthesia procedures for new patients to minimize the risk of postoperative kidney injury. Therefore, we divided the problem data into two categories based on the presence of kidney injury in patients and predicted the anesthesia measures during the operation in each group using a regression model. The low-risk and high-risk anesthesia approaches predicted by the two regression models are determined for each patient. The goal is to use low-risk values for the anesthesia parameters during the operation and consider a safety margin to avoid the high-risk values to minimize the risk of postoperative AKI.

## 3. Methods

### 3.1. An Overview of the Proposed Method

**Figure 1.**
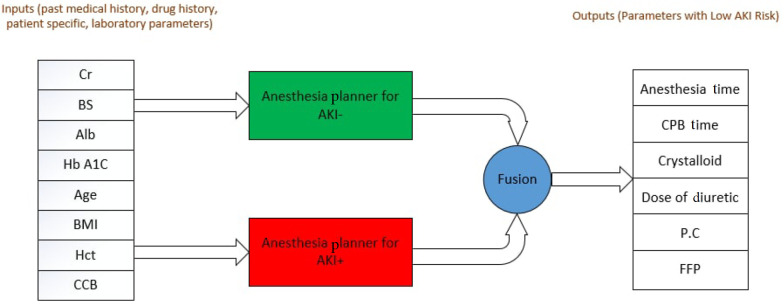
The Overall structure of the machine learning model for predicting optimal anesthesia parameters

The present study aims to design an intelligent assistant for cardiac anesthesiologists to determine appropriate intraoperative measures for anesthesia. Determining the intraoperative parameters of anesthesia is always challenging for physicians due to the long duration of cardiac surgery, its high sensitivity, and the possibility of postoperative kidney injury. This study proposed a model using machine learning techniques to determine intraoperative anesthesia parameters so that the patient suffers the least postoperative kidney injury. The patients were divided into AKI+ and AKI- groups to design this model. A regression model was developed and trained on data from each group individually. The presence of these two regression models allows the cardiac anesthesiologist to be aware of the optimal parameters of anesthesia and the high-risk values for these parameters to avoid their selection. Each model received the eight preoperative features of patients, including age, body mass index (BMI), use of calcium channel blockers (CCB), serum creatinine, serum albumin (Alb), blood sugar (BS), glycosylated hemoglobin (HbA_1_C), and hematocrit (Hct), which were recorded preoperatively as input to predict six critical parameters of intraoperative anesthesia. These parameters include cardiopulmonary bypass (CPB) time, anesthesia time, crystalloid dose, dose of diuretic, transfusion of packed red cells (PC), and fresh frozen plasma (FFP). The overall structure of the proposed model for reducing AKI risk is depicted in Figure 1. 

The proposed method consists of two separate regressions. After receiving information about the eight features of patients in the AKI- group, the first regression suggests six measures related to intraoperative anesthesia that present the lowest risk of AKI for the patient (Figure 1). In other words, when considering these six parameters during surgery, the possibility of AKI is minimized for the patient. The second regression supports the first by using data from the AKI+ group to establish a safety margin for deciding on anesthesia procedures. Essentially, this second regression aims to inform the anesthesiologist about the high-risk anesthesia procedures for AKI by learning from the AKI+ samples. By integrating the recommendations of these two regressions, the proposed model strives to suggest the least risky method of anesthesia for the patient. The main steps of the proposed method are explained in more detail below.

### 3.2. Data Collection

The data from 998 adult patients, including 598 men and 400 women who underwent open cardiac surgeries at Imam Hossein, Shahid Modarres, and Masih Daneshvari hospitals in Tehran, were included in this study. The exclusion criteria were non-adult patients. The final dataset included data from 526 patients with kidney injury after cardiac surgery and 472 patients without kidney injury, which were used in a ratio of 85 to 15 for training and testing data, respectively. According to the literature (20), the rules of thumb for machine learning studies suggest determining the sample size at 50 to 1000 times the number of predicted classes or 10 to 100 times the number of studied features. In addition to following these guidelines, the opinions of experienced anesthesiologists and pilot investigations have also been considered in determining the sample size.

### 3.3. Selecting Patients’ Preoperative Features

In the problem data for each patient, 27 different features were recorded, which constitute the patient’s preoperative data. These features are categorized into four groups: Patient demographics, past medical history (PMH), drug history, and laboratory features (Table 1). Due to the small volume of the problem data and the high number of patient features, building a machine learning model and training it with the data is challenging. Therefore, using the XGboost method (4), the authors identified the most important features for the present study, including serum creatinine (Cr), blood sugar (BS), albumin (Alb), glycosylated hemoglobin (HbA_1_C), age, body mass index (BMI), hematocrit (Hct), and calcium channel blockers (CCB).

### 3.4. Selecting Intraoperative Features of Anesthesia

The designed model aims to recommend specialist intraoperative anesthesia procedures to minimize postoperative kidney injury. Due to the limited amount of data, the XGboost method (4) and guidance from anesthesiologists were used to select the six most important anesthesia parameters for designing the machine learning model. These features include Anesthesia Time, CPB Time, Dose of Diuretic, Crystalloid Dose, and transfusion of Packed Cells (PC) and Fresh Frozen Plasma (FFP) (Table 2). 

**Table 2.** Anesthesia Parameters During Cardiac Surgery

Variables	Frequency (%)	Range	Mean ± SD
**Anesthesia time (min)**		130 - 160	346.81 ± 71.29
**Crystalloid dose (lit)**		1 - 2	1.49 ± 0.03
**Dose of diuretic (mg/dL)**		20 - 180	26.76 ± 3.04
**PC transfusion**			
Positive	582 (58.31)		
Negative	409 (40.98)		
Missing values	7 (0.71)		
**FFP transfusion**			
Positive	487 (48.87)		
Negative	506 (50.70)		
Missing values	5 (0.43)		
**CPB time (min)**		53 - 350	112.44 ± 32.33

Abbreviations: Anesthesia time, anesthesia duration in cardiac surgery; Crystalloid, amount of injected crystalloid drug during surgery; dose of Diuretic, two different dose of two different diuretics; PC, packed red cells; FFP, fresh frozen plasma; CPB time, cardiopulmonary bypass time.

### 3.5. Designing the Learning Model of Anesthesia Design Machine

The proposed model consists of three main components: AKI- Planner, AKI+ Planner, and AKI Risk Visualizer. The first two components are trained using AKI- and AKI+ samples, respectively. In the problem data, two labels, Cr-1 and Cr-7, indicate the patient's serum creatinine on the first and the seventh day after surgery, respectively. Using these values along with the serum creatinine level before surgery, postoperative injury is determined by an integer between zero and three, where zero indicates no injury and three indicates the highest level of injury. In this study, individuals who did not suffer any kidney injury on the first and the seventh day post-surgery were categorized in the AKI- group, while those who did were placed in the AKI+ group. The severity of kidney injury is determined based on the Kidney Disease Improving Global Outcomes (KDIGO) classification criterion (21), as detailed in Appendix 1. Each component is explained in detail in the following subsections.

### 3.6. AKI- Planner Component

This component utilizes eight important preoperative data points to determine six anesthesia parameters that minimize the patient's AKI risk. The AKI- Planner component is designed as a regression model using a three-layer neural network (Appendix 2). The output of the neural network corresponds to appropriate intraoperative anesthesia measures that carry the lowest risk of AKI. The AKI- Planner neural network is trained exclusively on data from members of the AKI- group. Consequently, the AKI- Planner aims to recommend to the anesthesiologist the optimal parameters to reduce the risk of AKI by analyzing the new patient’s features.

### 3.7. AKI+ Planner Component

This essential component has a structure similar to the AKI- Planner component. Like its counterpart, this component is a three-layer neural network with the same specifications (Appendix 2). Specifically, the AKI+ component predicts six high-risk anesthesia parameters of AKI by analyzing eight preoperative features of patients. This component supplements the first, providing critical information to the anesthesiologist. If the output from the AKI- Planner neural network is used solely in constructing the model, there is a risk that the anesthesia parameters proposed by the AKI- Planner may be close to values that carry a high risk of kidney injury. The AKI+ neural network is trained using data from 526 patients in the AKI+ group. Therefore, the output of the AKI+ Planner network should be considered in conjunction with the AKI- Planner network to avoid risky anesthesia parameters by determining the best anesthesia measures. This network is designed to assess the safety margin in decision-making for anesthesia procedures.

### 3.8. AKI Risk Visualizer Component

The AKI-Planner component determines six output parameters by receiving eight inputs before surgery and informs the anesthesiologist of the optimal anesthesia measures to reduce the risk of AKI. Additionally, this study aims to help anesthesiologists understand the intraoperative risk associated with selecting certain parameters. Consequently, software was developed to measure and compare the distance between the chosen anesthesia methods and the outputs of the AKI- Planner and AKI+ Planner models, as assessed by three expert cardiac anesthesiologists. Furthermore, the software plots these two distances on a graph, allowing the physician to assess the AKI risk associated with any of these values by examining the various settings and options of anesthesia procedures. The developed software is capable of performing the following activities:

(1) Receiving the patient’s information.

(2) Receiving the anesthesia parameters considered by the physician and applying changes to them.

(3) Displaying the proposed anesthesia parameters from the AKI- Planner model and the risks associated with selecting them.

(4) Displaying the AKI risk caused by selecting different anesthesia parameters.

(5) Calculating the distance from the anesthesia parameters considered by the physician to the outputs of the AKI- Planner and AKI+ Planner models.

**Figure 2.**
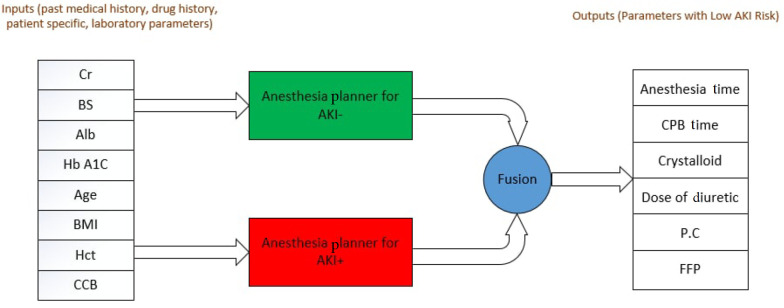
The environment of the developed simulator software

Figure 2 illustrates the environment of the developed software.

## 4. Results

### 4.1. Determining the Kidney Injury Label of Data Samples

In this section, the severity of postoperative kidney injury on the first and seventh days is determined (Appendix 1). Any patient who has suffered kidney injury on at least one of these days after surgery is placed in the AKI+ group. Conversely, patients who did not experience kidney injury on these days are placed in the AKI- group. Table 3 provides information on the dataset, including the number of training and test data.

**Table 3.** Dividing Dataset to the Train and Test Sets ^a^

Variables	No. of Samples	Training Set (85%)	Test Set (15%)
**Dataset**	998	848	150
**AKI-**	472 (47.29)	401	71
**AKI+**	526 (52.71)	447	79

^a^ Values are expressed as No. (%).

### 4.2. Experimental Results

**Figure 3.**
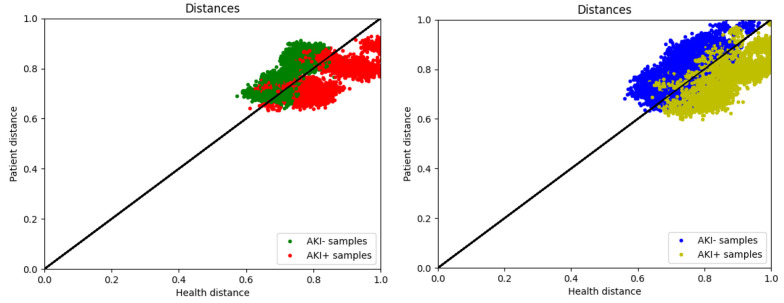
Predicting anesthesia parameters on A, test; and B, train data and comparison with the output of AKI+ and AKI- models

As mentioned in the previous section, the proposed method includes two models: The AKI+ Planner and the AKI- Planner. The AKI+ Planner model is trained based on the AKI+ group, while the AKI- Planner model is trained based on the AKI- group (Figure 3A). 

The data for the AKI+ group is often below the y = x line. In other words, the anesthesia parameters set by three expert cardiac anesthesiologists for individuals with postoperative kidney injury are closer to the output of the AKI+ model, indicating that this model accurately predicts high-risk AKI parameters. Conversely, the samples from the AKI- group are often above the y=x line, suggesting that most individuals without kidney injury are operated on with anesthesia parameters considered appropriate by the AKI- neural network. The results of the model prediction on training data are shown in Figure 3B, demonstrating the acceptable accuracy of the models in predicting anesthesia parameters. Additionally, Appendix 3 presents the results of the proposed models on the test dataset.

As illustrated in Figure 3A, the green and red points represent samples from the AKI- and AKI+ groups, respectively. To evaluate the accuracy of the model, samples were assumed to be predicted negative above the y = x line and predicted positive below the y = x line. Appendices 4 and 5 display the confusion matrix of the designed models on the training and test data.

Table 4 also presents the performance evaluation metrics of the designed model on the dataset.

**Table 4.** Evaluation Metrics for the Model on Train and Test Set

Variables	Precision	Recall	Accuracy	F1-Score
**Train set**	0.806	0.785	0.787	0.795
**Test set**	0.797	0.848	0.806	0.821

The best performance of the models occurred when the AKI- network predicted the largest number of samples above the y = x line, and the AKI+ network predicted the largest number of samples below the same line. Consequently, an accuracy of 80.6% was achieved in measuring the performance of the models.

### 4.3. Evaluation of the Proposed Method by Anesthesiologists

To evaluate the output quality of the proposed models, a study was organized for cardiac anesthesiologists by presenting the features of 60 patients undergoing cardiac surgery alongside three different approaches. The study involved three expert cardiac anesthesiologists: A professor with over 20 years of experience in research and treatment, an assistant professor with over 15 years of experience, and a cardiac anesthesiologist with three years of experience in the field. The specialists were asked to select one of the three treatment methods for each patient based on the parameters related to the patient's medical and pharmacological history. Thirty of these patients had postoperative AKI, while the other 30 did not suffer any injuries. To determine the treatment options presented to the specialists, we clustered the patients according to their intraoperative anesthesia parameters. Three clusters were then selected: Two containing low-risk anesthesia parameters and one containing high-risk parameters. The k-means clustering method was used to determine these clusters (22, 23). The features of the three cluster centers, designed as survey options, are depicted in Table 5. 

**Table 5.** The Candidate Treatment Methods by Running K-means Clustering Algorithm on Anesthesia Parameters

Variables	Treatment Method 1	Treatment Method 2	Treatment Method 3
**Anesthesia time (min)**	340 - 350	360 - 400	330 - 340
**CPB time (min)**	110 - 120	130 - 140	100 - 110
**Dose of diuretic furosemide (milligram)**	20 - 40	40 - 60	20-30
**Crystalloid dose (liter)**	1 - 2	2 - 3	1 - 2
**Pc**	Received	Not Received	Received
**Ffp**	Not Received	Received	Received

Since three specialists were involved, the majority vote was considered for each patient as their proposed treatment. Additionally, 30 patients were in the AKI+ group, while the other 30 were in the AKI- group. For each patient, the specialists' majority vote was determined, and the suggested treatment option was classified as belonging to either the AKI+ or AKI- groups based on this vote. A confusion matrix can be presented by considering the patient's postoperative kidney injury condition and the specialists' recommended treatment (Appendix 6).

**Figure 4.**
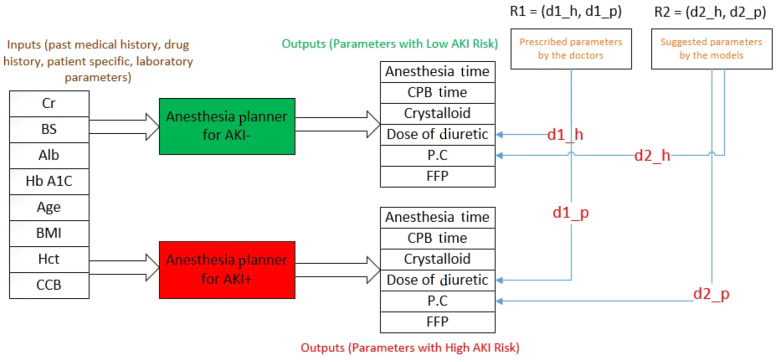
Comparing the risk of anesthesia parameters determined for surgery using the proposed parameters by the model

The distance between the anesthesia parameters in the patient data file and the output of the two designed planners was calculated. Subsequently, a point in the coordinate space was obtained, indicating the accuracy of the developed models. The accuracy of the designed model is higher in determining the appropriate intraoperative parameters of anesthesia as the point rises above the y=x line. A method was then proposed to measure the model's efficiency. For each patient, six parameters of intraoperative anesthesia, representing the majority vote of the specialists, were selected. The distance between this vector and the output of the two AKI+ and AKI- Planner components was measured. The position of the new point on the coordinate screen was then compared with that of the previous point, indicating the distance between the treatment the patient received and the output of the AKI+ and AKI- Planner models. If the point shifted to the area above the y = x line, the proposed model's predicted values for anesthesia parameters were more accurate and carried a lower AKI risk compared to the method with which the patient was anesthetized. Figure 4 presents a schematic of the proposed method. As shown, the points R1 = (d1_h, d1_p) and R2 = (d2_h, d2_p) represent the distance between the performed treatment and the proposed treatment of the model with the output of the two regressions, respectively.

Appendix 7 displays the flowchart for the movement of points on the screen. Blue points represent test data that turned green after applying the specialists’ evaluations on anesthesia parameters. Furthermore, most of the points moved above the y = x line, indicating the acceptable accuracy of the model for use as an intelligent assistant by anesthesiologists.

### 4.4. Anesthesia Management Recommender Software

The software functions such that an anesthesiologist can view the AKI risk associated with selecting certain parameters on the AKI plot when they enter patient information, including medical and pharmacological records along with intraoperative anesthesia parameters, into the software environment (Appendix 8). Additionally, data from several patients with medical and pharmacological records similar to the current patient are displayed on the AKI plot. This allows the specialist to intuitively understand the intraoperative anesthesia parameters, observe which anesthesia parameters were used for patients similar to the current one, and be aware of their surgery AKI risk. This software features a slider for each anesthesia parameter, allowing adjustments to the parameter values. Appendices 7 and 5 present information about one of the patients and his AKI risk due to the selection of different anesthesia parameter values, represented by a blue point. Moreover, the blue point moves within the coordinate space as the parameter values are adjusted.

**Figure 5.**
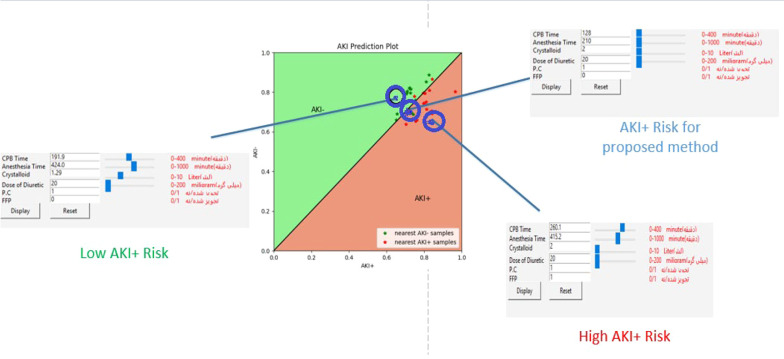
Changes in the risk of AKI due to choosing different set of values for anesthesia parameters

The specialist can manipulate the blue point representing the AKI risk so that it reaches above the y = x line by evaluating different settings of anesthesia parameters. In this scenario, the intraoperative anesthesia parameters carry a relatively low risk of AKI. Figure 5 illustrates a group of anesthesia parameters that the patient underwent, categorized under the AKI+ risk by the proposed method. The AKI risk of selecting these values is represented by a point near the y = x line, indicating that the chosen values for the intraoperative anesthesia parameters are not considered risky. However, due to the presence of many red points around this one, it cannot be deemed low risk. By changing the values of the anesthesia parameters, the AKI risk changes, and the blue point on the chart shifts. Two different boxes are highlighted in red and green (Figure 5). The high AKI+ risk box indicates the high-risk values of the intraoperative anesthesia parameters, selecting which causes the blue point to move below the y = x line. Consequently, using these values will increase the risk of postoperative AKI. Conversely, the Low AKI+ Risk box displays the low-risk values for the anesthesia parameters. In other words, if the desired blue point is above the y = x line after altering the intraoperative anesthesia parameters, the specified anesthesia parameters are considered low-risk and can reduce the postoperative AKI risk.

## 5. Discussion

Cardiac surgeries are associated with postoperative AKI for various reasons. AKI may occur due to reduced kidney perfusion pressure after the patient is connected to a cardiopulmonary bypass pump. Other contributing factors include changes in kidney vascular tone, activation of the systemic inflammatory response, blood contact with the artificial surfaces of the bypass circuit, and the production of microemboli, which can result in AKI, potentially leading to the need for dialysis. The incidence of AKI following cardiac surgery is 30%, while the average rate of dialysis in patients after these surgeries is 4% (5). The long-term consequences of AKI after cardiac surgery include chronic kidney failure, decreased quality of life, increased risk of cardiovascular incidents, and increased mortality rates, such that even very low levels of increase in serum creatinine (0.3-0.5 mg/dL) are associated with a marked increase in 30-day mortality. Using renal replacement therapy (RRT) in the postoperative period is one method that increases the hospital survival rate of patients with AKI (5). However, AKI requiring RRT is associated with a 50% mortality rate (O’Neal, Shaw, & Billings, 2016). Additionally, this method increases the cost of the patient's treatment by an average of $3629 per year (24). As mentioned in previous sections regarding the costs of hospitalization and treatment of patients with kidney injury, the cost imposed on these patients accounts for a significant percentage of the healthcare budget in each country (25).

Beyond the medical and treatment costs, there are various costs in the medical education department for training cardiac anesthesiologists. Despite using their skills during anesthesia in heart surgery, there is always a high risk of postoperative kidney injury. Designing an intelligent assistant to determine the intraoperative parameters of anesthesia can significantly advance their medical goals and reduce various costs, including those associated with postoperative treatment, kidney injury in the patient, and the training and education of specialists.

### 5.1. Conclusions

In the present study, a model based on machine learning techniques was developed to inform anesthesiologists of the AKI risk associated with different methods of intraoperative anesthesia. This model comprises the AKI+ Planner, AKI- Planner, and AKI Risk Visualizer, each designed to identify optimal and high-risk anesthesia parameters. The components of the AKI+ Planner and AKI- Planner were separately trained on AKI+ and AKI- samples, achieving accuracies of 78.7% and 80.6% on the training and test data, respectively. These results indicate the acceptable performance of the proposed method. Additionally, a multiple-choice questionnaire was designed to more accurately gauge the model's performance, during which expert anesthesiologists recommended treatments for 60 patients. This process demonstrated that the developed model predicts optimal surgical parameters with higher accuracy when comparing the specialists' opinions with the treatments actually administered to patients.

Furthermore, software was developed to serve as an intelligent assistant for anesthesiologists in determining intraoperative anesthesia parameters. This tool allows them to evaluate various anesthesia parameters and assess the AKI risk. In future studies, the authors plan to enhance the accuracy of the designed model by expanding the dataset and the team of experts. Finally, other methods could be explored to combine the results of the two regression models and further improve the proposed method.

aapm-14-3-143853-s001.pdf

## Data Availability

The dataset presented in the study is available on request from the corresponding author. The data are not publicly available due to some organizational considerations.
