# Supplementary material for: Anesthetic Management Recommendations Using a Machine Learning Algorithm to Reduce the Risk of Acute Kidney Injury After Cardiac Surgeries
Source: Anesth Pain Med. 2024 Jun 5;14(3):e143853. doi: 10.5812/aapm-143853 (PMC11474233; doi:10.5812/aapm-143853)
Supplement: aapm-14-3-143853-s001.pdf [file aapm-14-3-143853-s001.pdf]

Appendix 1: Determining the severity of kidney injury

Status Description	Kidney injury severity level
(Cr7 – Cr < 0.3 and Cr1 – Cr < 0.3)<br>or<br>(Cr7/Cr < 1.5 and Cr1/Cr < 1.5)	No injury
(Cr1 – Cr = 0.3 or Cr7 – Cr = 0.3)<br>or<br>(1.5 < Cr1/Cr < 1.9 or 1.5 < Cr7/Cr < 1.9)	Stage 1
2 < Cr1/Cr < 2.9 or 2 < Cr7/Cr < 2.9	Stage 2
(Cr1 – Cr > 4 or Cr7 – Cr > 4)<br>or<br>(Cr/Cr > 3 or Cr7 /Cr > 3)	Stage 3

Appendix 2: Neural network parameters designed for AKI- regressor

Layer (type)	Output Shape	#Param	Activation
dense (Dense)	(None, 14)	126	RELU
dense_1 (Dense)	(None, 8)	120	RELU
dense_2 (Dense)	(None, 6)	54	Sigmoid

Appendix 3: Error rate for AKI- Planner, AKI+ Planner on AKI-, AKI+ dataset

	**AKI- Test samples**	**AKI+ Test samples**	**AKI- Train samples**	**AKI+ Train samples**
**RMSE**	0.0906	0.0887	0.1074	0.1067

Appendix 4: Confusion Matrix for neural network evaluation on train data

	Real Labels	
**Predicted Labels**	**Positive**	**Negative**
**Positive**	True Positive (TP)<br>351 samples (41.39%)	False Positive (FP)<br>84 samples (9.91%)
**Negative**	False Negative (FN)<br>96 samples (11.32%)	True Negative<br>317 samples (31.38%)

Appendix 5: Confusion Matrix for neural network evaluation on test data

	Real Labels	
**Predicted Labels**	**Positive**	**Negative**
**Positive**	True Positive (TP)<br>67 samples (44.67%)	False Positive (FP)<br>17 samples (11.33%)
**Negative**	False Negative (FN)<br>12 samples (8.00%)	True Negative<br>54 samples (36.00%)

Appendix 6: Confusion Matrix for survey result

	Real Labels	
**Predicted Labels**	**Positive**	**Negative**
**Positive**	True Positive (TP)<br>9 samples (15.00%)	False Positive (FP)<br>3 samples (5.00%)
**Negative**	False Negative (FN)<br>21 samples (35%)	True Negative<br>27 samples (45%)

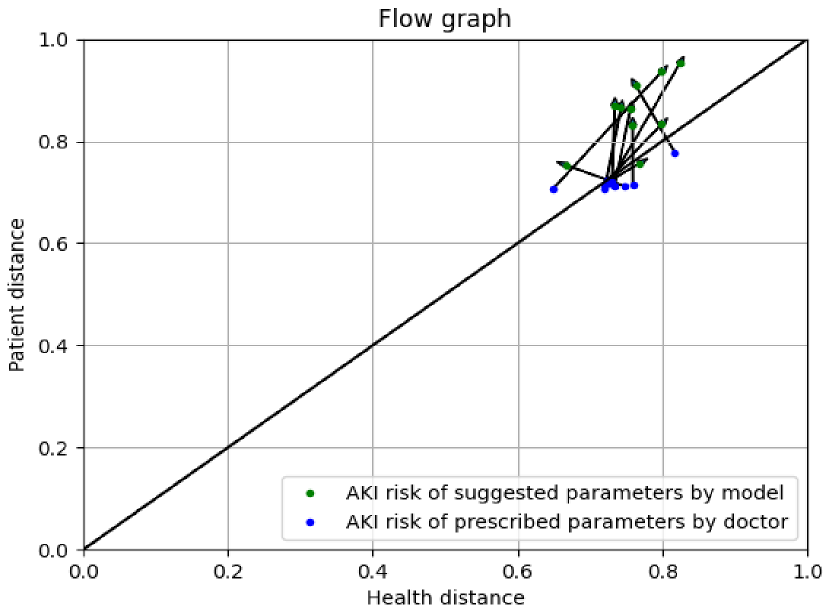

Appendix 7: The flow graph indicating movement of points in the space.

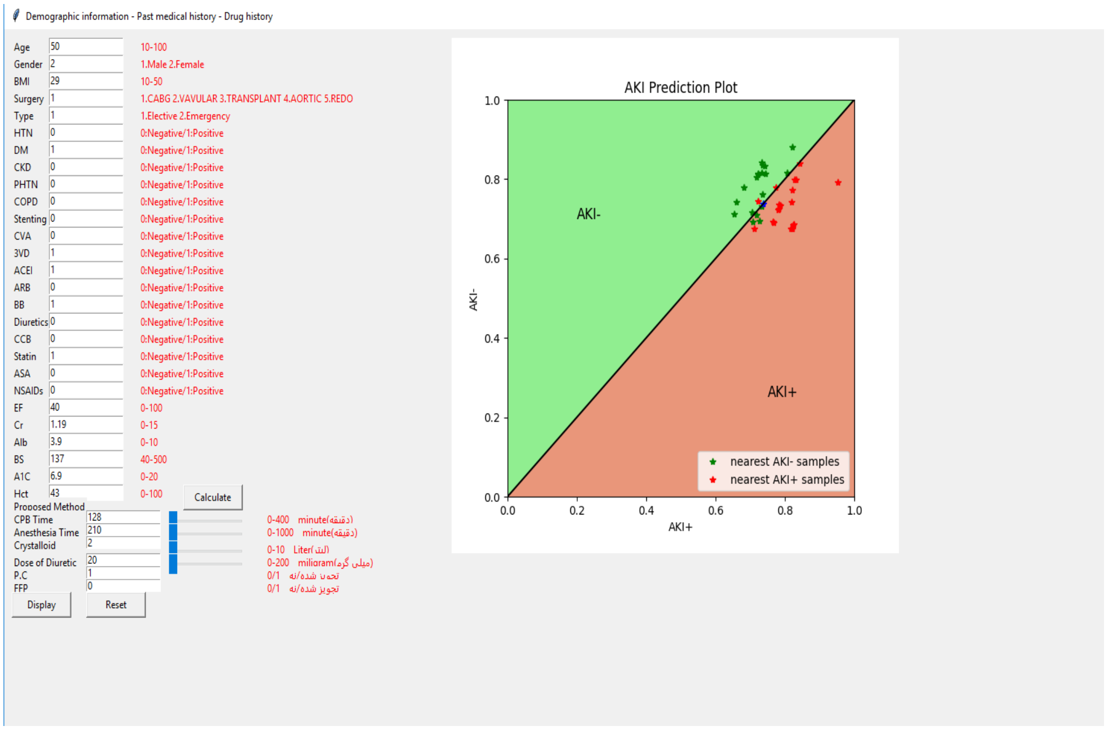

Appendix 8: The simulator software for evaluating AKI risk of anesthesia parameters.
